# A Reconfigurable Real-Time Compressive-Sampling Camera for Biological Applications

**DOI:** 10.1371/journal.pone.0026306

**Published:** 2011-10-18

**Authors:** Bo Fu, Mark C. Pitter, Noah A. Russell

**Affiliations:** Institute of Biophysics, Imaging and Optical Science, Schools of Biology and Electrical and Electronic Engineering, The University of Nottingham, Nottingham, United Kingdom; Friedrich-Schiller-University Jena, Germany

## Abstract

Many applications in biology, such as long-term functional imaging of neural and cardiac systems, require continuous high-speed imaging. This is typically not possible, however, using commercially available systems. The frame rate and the recording time of high-speed cameras are limited by the digitization rate and the capacity of on-camera memory. Further restrictions are often imposed by the limited bandwidth of the data link to the host computer. Even if the system bandwidth is not a limiting factor, continuous high-speed acquisition results in very large volumes of data that are difficult to handle, particularly when real-time analysis is required. In response to this issue many cameras allow a predetermined, rectangular region of interest (ROI) to be sampled, however this approach lacks flexibility and is blind to the image region outside of the ROI. We have addressed this problem by building a camera system using a randomly-addressable CMOS sensor. The camera has a low bandwidth, but is able to capture continuous high-speed images of an arbitrarily defined ROI, using most of the available bandwidth, while simultaneously acquiring low-speed, full frame images using the remaining bandwidth. In addition, the camera is able to use the full-frame information to recalculate the positions of targets and update the high-speed ROIs without interrupting acquisition. In this way the camera is capable of imaging moving targets at high-speed while simultaneously imaging the whole frame at a lower speed. We have used this camera system to monitor the heartbeat and blood cell flow of a water flea (*Daphnia*) at frame rates in excess of 1500 fps.

## Introduction

High-speed cameras are widely used in scientific research in areas such as metrology [1], applied physics [2] and biology [3]. Many high-speed imaging applications in biological research concern the study of excitable, neural [4] and cardiac [5], cells. In these applications it is desirable to image cells at very high frame rates (>1 kfps) for extended periods; preferably indefinitely. This is typically not possible however because the system bandwidth imposes a hard limit to high-speed imaging. The bandwidth, which is defined by the maximum rate of data conversion and transfer to storage media, limits the number of pixels acquired per second. A trade-off between the frame rate and the spatial resolution is therefore always necessary. Analogue-to-digital conversion and on-camera memory can be very fast (GHz) and so the system bottleneck is often the data transmission link from the camera to its host computer. Even when the system bandwidth is not a limiting factor, continuous high-speed acquisition results in very large volumes of data that are difficult to handle, particularly when real-time analysis is required.

To partially overcome this bandwidth limitation images can be temporarily stored on the camera itself. Using this technique Kitamura *et al.*, for example, developed a high-speed camera with a 300,000-pixel single CCD in 2007 [6]. This camera is capable of running at 1,000,000 fps and storing 144 frames with an external memory. The number of recorded frames was improved to 288 frames by the authors in 2008 [7]. To enable continuous imaging a rolling buffer can be employed. For example Karimov *et al.* have designed a high-speed camera with a pre-trigger system [8]. The rolling buffer stores the most recent 103 frames at 1 Mfps. When an event is detected a trigger stops writing to the buffer so the frames can be saved. However, this still requires the data to be downloaded over a limited bandwidth link. Commercial cameras typically adopt this approach and use on-camera storage for high-speed imaging. Many neural and cardiac applications have successfully utilised such cameras, for example; Bullen *et al.* used a high-speed commercial camera (WV-1500, Panasonic) to develop a high-speed, random-access, laser-scanning microscope to record fast physiological signals from small neuronal structures [9]; the NeuroCCD-SM camera (Redshirt Imaging) has been used by Obaid *et al.* with potentiometric dyes to analyze neural networks [10]; and a high-speed camera CA-D1_A (Dalsa Inc.) has been used by Agronskaia *et al.* to build a fast time-domain based fluorescence lifetime imaging (FLIM) system [11]. These cameras are all able to run at high speed but only for short durations. The finite size of storage space available on the camera means this approach will always be unsuitable for applications that require continuous recording and real-time data analysis.

Instead of storing the data on the camera for later transmission the volume of data can be reduced by acquiring fewer pixels within each frame in a limited region of interest (ROI). In this approach only those pixels which contain useful information are digitized in order to reduce the volume of acquired data and make the acquisition faster. Graetzel *et al.* used this approach to analyse wing kinematics on a tethered *Drosophila* with a commercial CMOS camera (MV-D1024E-80-CL from Photonfocus AG Company) [12]. They initially ran the camera in full-field mode to determine the ROI and then set the camera to ROI mode to acquire smaller images at a very high frame-rate (approximate 1,500 fps) to allow real-time image analysis. This allowed them to use the available bandwidth efficiently. However, the size and the position of the ROI had to be defined *a priori* and could not be changed while high-speed imaging was taking place. Thus, even if the target remains within the camera's field-of-view, if it moves out of the small ROI, the recording will fail.

An alternative strategy that utilizes the limited bandwidth more efficiently is to reduce the volume of data to be transferred by compressing it first. For example, Chan *et al.* have developed a real-time compression system which was capable of recording images at 500 fps continuously [13]. They placed a compression circuit, implemented on a field-programmable-gate-array (FPGA), between the camera and the frame grabber. Although this method makes efficient use of the available bandwidth such a hardware based approach is complex to implement.

Recent work has investigated the concept of compressive sensing for imaging applications. Compressive sensing is similar in concept to image compression except that it reduces the number of samples acquired rather than remove redundancy from the image post-acquisition. In an example of this Robucci *et al* describe an imaging system that uses a custom sensor to perform ‘noiselet’ transforms (a form of pseudo-random wavelet transform) during image acquisition [14]. This is an interesting approach, but it requires specialist sensor hardware that is not yet commercially available and primarily concentrates on image, rather than video, acquisition.

In this paper, we present a camera system that attempts to overcome the limitations of these strategies by utilizing a form of compressed imaging. The camera system adaptively concentrates most of the available bandwidth on a subset of pixels while sampling the remainder of the pixels at low-speed. In this way the camera is able to use most of its available bandwidth to capture images of the ROI at a relatively high-speed while simultaneously acquiring high-resolution images at low-speed using the rest of the bandwidth. In addition, the camera is able to use this full-frame information to calculate the positions of targets and update the high-speed ROIs without interrupting acquisition. This allows the camera to track and image moving targets at high-speed while simultaneously imaging the whole frame at a much lower frame rate. While the overall bandwidth of the camera is low it is utilised efficiently and this allows changing ROIs to be acquired continuously at high speed. Because the total volume of data is reduced considerably by only collecting the necessary information at the source, pressure is reduced on down-stream systems; including real-time data analysis.

Efficient detection of light tends to be critical in high-speed imaging applications. Many cameras, however, cannot acquire photons during the readout phase and this results in dead-time during which valuable signal is lost. Our camera has the additional advantage of having virtually no dead-time as it is able to detect photons continuously. This is possible because the CMOS sensor has multiple channels per pixel; allowing the exposure and readout of two channels to be alternated. This is similar in concept, but more flexible in operation than an interline CCD.

The camera system was built using; a custom-made multi-channel CMOS sensor chip capable of arbitrary addressing; a data acquisition (DAQ) board; a computer running Microsoft Windows; and custom software written in LabVIEW. The hardware and software architecture implemented in this reconfigurable real-time feedback-control system is simple and flexible and could easily be applied to other devices. It is immediately applicable, for example, to CMOS based multi-electrode array devices; which are also used to monitor the activity of neural networks and cardiac myocytes [15] [16]. The system design allows it to be easily integrated with motorized stages, spatial light modulators (SLM) and other devices.

## Materials and Methods

### Hardware

**Figure 1:**
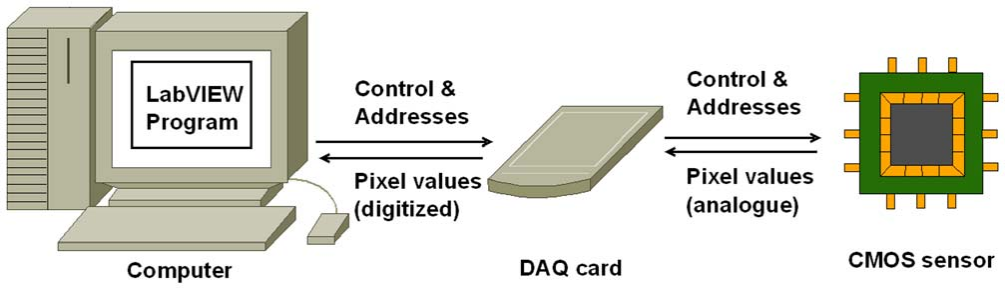
Camera system design. The camera system consists of a custom made CMOS sensor chip, a data acquisition card (DAQ) and a computer with camera software programmed in LabVIEW. The DAQ card generates digital control and address signals for the CMOS sensor and synchronously acquires the raw analogue signals directly from each pixel. All of the camera functionality is implemented in the LabVIEW program on the computer.

The camera system, comprising of both hardware and software, is illustrated in Figure 1. The entire system consists of a personal computer, a data acquisition (DAQ) card, (NI USB 6259) and a CMOS sensor (A64P). Software on the computer generates digital control signals, including the reset, shutter and the addressing, and transmits these to the sensor via the DAQ card. The DAQ card then synchronously acquires the analogue voltage from the corresponding pixel and converts it to a digital signal. Synchronization of the digital output and the analogue input tasks is achieved on the DAQ card - using its counter as the base clock.

The entire functionality of the camera including; acquisition, analysis, display, feedback control, and storage of the images, are performed on the computer by a custom program written in LabVIEW software.

#### CMOS Sensor

**Figure 2:**
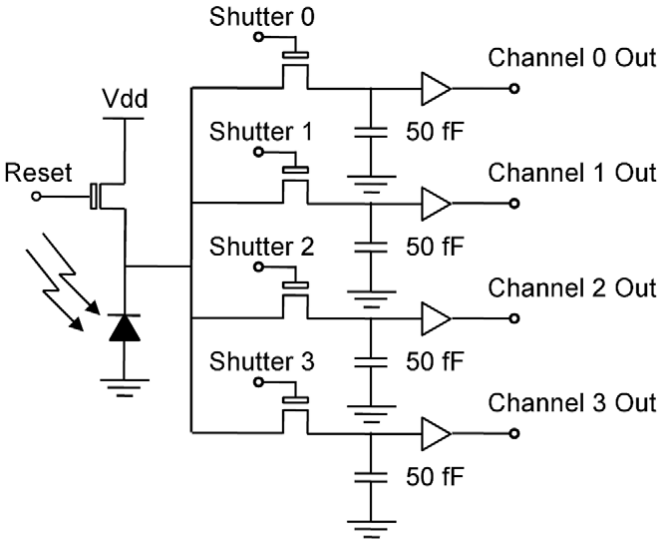
Single pixel design. Schematic of a single pixel on the A64P CMOS sensor. The sensor contains a four channel standard active-pixel sensor (APS) architecture with a global shutter. During exposure the reset switch is open and the shutter on one channel is closed. This shutter is then opened until the voltage has been read off the storage capacitor. The four channels can be read out independently. This chip design allows read-out and exposure to occur on different channels simultaneously.

The camera sensor chosen to carry out this work was a custom-made CMOS 64×64 pixel array (denoted A64P) [17]. This sensor (Figure 2) has a standard active-pixel sensor (APS) architecture [18] with a global shutter. It was selected for two main reasons: first, the sensor is arbitrarily addressable and access to the full address bus is available; second, the camera has four independently shuttered capacitors in each pixel. By using two, or more, of these capacitors light integration and readout can be carried out simultaneously to virtually eliminate dead-time.

As with standard global shutter APS designs the entire pixel array on sensor chip A64P is simultaneously reset by a single digital pulse. The exposure then begins when this reset pulse ends and finishes when the shutter switch is opened. During exposure the incident light level is stored as a voltage on the pixel's capacitor. Once exposure is completed the analogue voltage stored in each pixel can be randomly accessed by placing the appropriate word on the address lines. It is possible, therefore, to read any arbitrary subset of the array before starting the next exposure. This is in contrast to a CCD sensor where the entire image (or contiguous blocks at least) must be read out sequentially. The A64P sensor is able to output four parallel channels of data at up to 10 MHz per channel [17] [15].

### Software

To allow the system to be used flexibly by a wide range of end users the camera software was coded using LabVIEW from National Instruments. LabVIEW is a data-flow based high-level, graphical, programming language. LabVIEW has allowed us to develop a camera system with all of the functionality defined in software that can be easily replaced without modifying the hardware.

#### Operating Modes

The software was designed in a modular format to allow the camera to operate in a variety of modes depending on the demands of the application. The basic operating mode is continuous single-channel full-frame imaging. This can then be extended to dual-channel mode in order to eliminate dead-time by using two channels per pixel and alternating exposure and read-out. As the camera has four channels in total it is also able to operate in double-sampling mode. In double-sampling mode the shutter of one channel is closed, allowing the capacitor to discharge during exposure, while the shutter on a second channel is left open. This allows the pixels to capture a dark frame simultaneously with the exposed frame. The four channels available on the A64P sensor allows double-sampling mode to be implemented in either single-channel or dual-channel operation.

For applications demanding higher imaging speeds an ROI mode can be applied with arbitrary size and shape. Because the system bandwidth is defined by the pixel sampling-rate the frame-rate in ROI mode can be increased by decreasing the size of the ROI. The ROI mode can then be extended to a high-speed ROI/low-speed full-frame mode in which an arbitrary fraction of the bandwidth is sacrificed to acquire full-frame images. In this mode most of the available bandwidth is used to image the ROI at high-speed while the rest of the bandwidth is used to scan the whole field at a lower frame rate. As a special case of this operating mode the camera is able to capture low-resolution (subsampled) images of the full field at high-speed, while simultaneously acquiring high-resolution images at low-speed. This mode can be achieved by defining the ROI as a subsampling matrix and has similarities in operation to [19]. Finally, a tracking-ROI mode has also been implemented in which the full-frame information is used to calculate the positions of targets and the high-speed ROIs are updated without interrupting acquisition. This allows the camera to image moving targets at high-speed while simultaneously imaging the whole frame at a lower speed. All of these modes can be implemented in single-channel or dual-channel mode; with or without double-sampling.

#### Software Architecture

**Figure 3:**
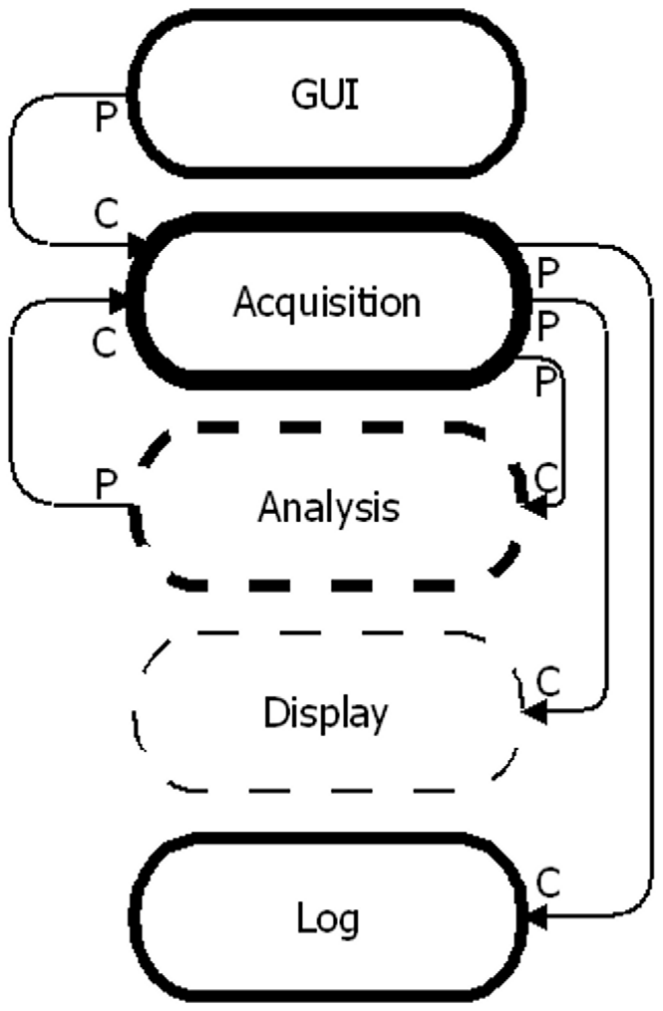
Software design. Example schematic of the software. The arrows show data flow from producer loops (P) to consumer loops (C). Solid loops represent lossless data process while dotted loops represent lossy process. For example, commands from the user are processed by the Graphical User Interface (GUI) producer loop before being passed, without loss, to the Acquisition consumer loop. Note that loops can be both producers and consumers (e.g. Acquisition). The line thickness of the loop represents its running priority. Thicker lines indicate higher priority tasks.

In order to implement a modular camera, which is both robust and as versatile as possible, Queued State Machine-Producer/Consumer Loop (QSM-PC) architecture was used (Figure 3). This architecture decouples tasks so that they can run independently from each other. It allows many tasks to be run in parallel with different, assigned, priorities. Tasks including; user interface, data acquisition, data analysis, display and data logging, were each assigned their own loop.

In a QSM-PC based program each loop (task) packages the data and labels it before it is passed from the “producer loop” (the loop that generates the data) to the “consumer loop” (the loop that handles the data). The consumer loop then processes this data package according to the label attached to it. The data generated by a producer loop is temporarily stored in a first-in-first-out (FIFO) buffer until the consumer loop is ready for it. Because the consumer loop is not interrupted the QSM-PC architecture allows us to update the sensor commands, adaptively, without interrupting acquisition. It is worth noting that any loop is also able to act both as a producer loop and a consumer loop.

The FIFO buffer can be lossless or lossy according to the requirements of the consumer loop. In some situations a loss of data is desirable in which case the buffer is simply overwritten. For example, when acquiring images at 10000 fps they can only be displayed on the monitor at 60 fps. In this case the buffer is continuously overwritten and the consumer loop grabs the latest data when it is ready.

**Figure 4:**
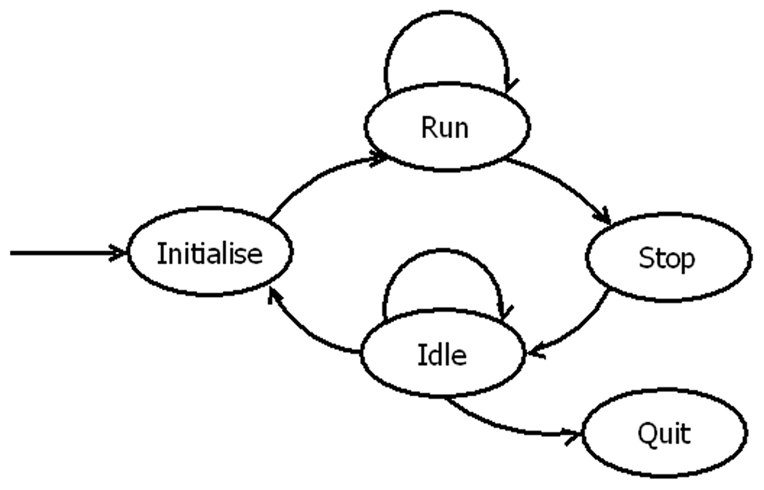
Example finite state machine. Schematic of the Acquisition loop finite state machine (FSM). At all times the system exists in a state (ovals) and when the appropriate condition is met the system changes (arrows) to another state. Using state-machine architecture allows for error conditions to be managed and for the code to be designed in a flexible and robust manner.

Within each loop a finite state machine was implemented; an example of which is shown in Figure 4. State machines are robust, flexible and easy to maintain. The functionality of the system can be modified easily by adding or modifying the states.

### Camera Operation

#### Basic Operation

**Figure 5:**
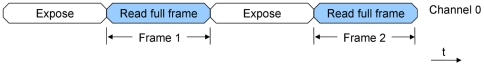
Single-channel mode. The sequence of commands during single-channel mode. This is the basic mode of operation for the camera system in which a single channel is used to first expose and then read out images sequentially.

During basic imaging the sensor is first exposed to light and then, for each pixel, the voltage on one of the four storage capacitors (channel 0) is read out pixel-by-pixel until the full frame has been acquired (Figure 5). Since the charge on the capacitor must be held until read-out has been completed the sensor cannot be exposed to light during this time. This period is the dead-time and results in an unacceptable waste of photons in high speed imaging applications. The dynamic range of this camera is 57 dB.

#### Dual-channel sampling

**Figure 6:**
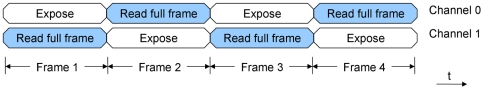
Dual-channel mode. The sequence of commands during dual-channel mode. In this mode the camera uses two channels with the exposure and readout alternating between channels. This virtually eliminates dead-time.

Dead-time can be virtually eliminated by operating in dual-channel mode. Two channels per pixel are used with alternating exposure and read-out (Figure 6). So, while one channel is being read out, the other is capturing light. Because the camera is continuously exposed dead-time is virtually eliminated.

The chip A64P has four channels per pixel so it is also possible to implement double sampling in both single-channel and dual-channel modes. During double sampling the shutter of one channel is closed, allowing the capacitor to discharge during exposure as normal, while the shutter on a second channel is left open. This allows a dark frame to be captured simultaneously with the exposed frame.

#### High-speed ROI

**Figure 7:**
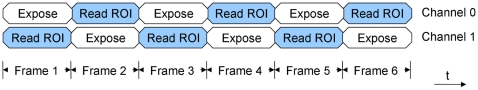
ROI mode. The sequence of commands during ROI mode. In this mode the frame rate of the camera can be increased by reading only regions of interest (ROIs). ROIs of arbitrary size and position can be selected interactively using the graphical user interface. Higher frame rates can be achieved by selecting smaller ROIs.

The bottleneck of data transmission in our system is determined by the rate of the analogue to digital convertor (ADC) (1 MS/s). Although this could easily be improved by using a faster ADC it is sufficient to demonstrate high-speed imaging that utilizes the available bandwidth most efficiently. To do this we can read a small ROI instead of the whole image (Figure 7). The locations of any arbitrary set of ROIs can be set, interactively, using the graphical user interface.

#### High-speed ROI with low-speed full-frame

**Figure 8:**
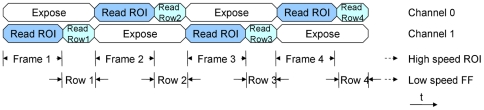
ROI plus full-frame mode. The sequence of commands during the “high-speed ROI with low-speed full-frame” imaging mode. In this mode the bandwidth of the camera system can be arbitrarily allocated. In this example, during each ROI acquisition a single row from the whole field is also captured. The full frame is then built up at low speed while the ROI is simultaneously acquired at high speed (64 times faster in this case).

While the camera is capturing the ROI at high speed it can also scan the background slowly. There are various possible strategies to achieve this, however, for simplicity we choose the approach shown in Figure 8.

During the first readout period both the ROI and the first full row are read. In the next readout period the same ROI is read again and the full second row. After the camera has acquired all 64 rows it combines them into a full-frame image. The ROI images and the full-frame images are written to disk separately so they can be played back and analyzed independently. As a special case of this mode of operation the ROI can be set to sub-sample the full-frame. Every fourth pixel, for example, could be included in the ROI; resulting in a 16×16 image acquired at 64 times the frame-rate of the full 64×64 image. Other strategies are possible that also achieve this but use the bandwidth more efficiently.

#### Tracking high-speed ROI with low-speed full-frame

**Figure 9:**
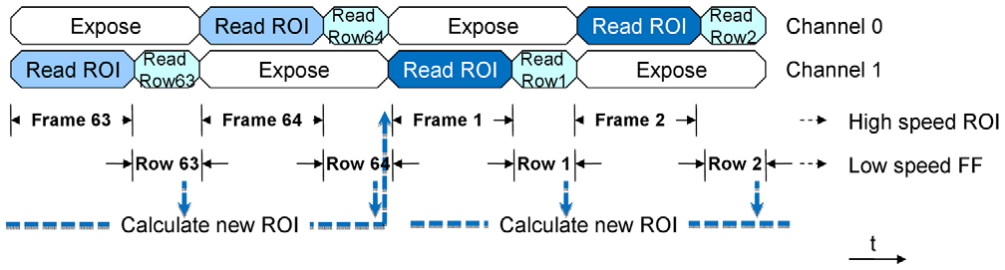
ROI tracking mode. The sequence of commands during the tracking mode. This mode operates the same as the “high-speed ROI with low-speed full frame” mode except that after acquiring a full frame the image is processed to determine where the interesting regions in the image are. If they have moved then the ROI is then updated by sending a new set of addresses to the acquisition loop without interrupting image capture. In this way moving targets at able to be imaged with high-speed ROIs while simultaneously capturing low-speed full-frame images.

Finally, after simultaneously acquiring the ROI at high speed and the full-frame at a lower speed, the full-frame image can be analyzed to determine the most appropriate ROI (Figure 9). The Analysis loop can then pass any changes to the ROI addresses to the Acquisition loop. The Acquisition loop gets the most recent set of ROI addresses from the FIFO buffer when it is ready and so continuous acquisition is not interrupted. In this way moving targets can be tracked.

To demonstrate this principle we implemented the simplest possible algorithm. Additional algorithms, suited to each application, can easily be developed and incorporated into the software. In this algorithm we assume the unchanging regions in the sample are not of interest; whereas any changing pixels in the sequence of images define the target that we wish to track. The changing regions were determined by subtracting subsequent full-frames in order to get a sequence of differential images. The number of ROI pixels (N_ROI_) was predefined and the ROI was defined to be the N_ROI_ largest pixels from the differential image (i.e. the N_ROI_ pixels that changed the most). Because the ROI is determined by changes in the image, rather than a fixed area, the camera can track multiple targets and will automatically adjust the ROI when a target enters or exits the camera's field-of-view.

Note that there is a latency between when the camera first identifies a new set of ROI addresses and when the ROI is updated. This occurs because the DAQ card has a hardware buffer, which is required to guarantee deterministic operation when running a non-deterministic operating system; such as Microsoft Windows. New addresses that are added to this FIFO buffer must work their way through the queue before they take effect. The size of this buffer is approximately 40,000 samples so at 1 MS/s it takes about 40 ms to update the ROI. Since the ROI is not updated every frame it was set to cover an area slightly larger than the calculated ROI to ensure that it does not lose track of the target.

## Results

### Experimental setup

To demonstrate the camera's utility in a biological application we imaged the heart and blood cells of a live *Daphnia* (water flee). *Daphnia* are mostly transparent and their rapidly beating heart and moving blood cells make them an ideal preparation for studying basic cardiac physiology. As *Daphnia* are nearly transparent the contrast in the raw images was poor. We have therefore presented both the differential images and the raw images to illustrate the targets more clearly.

**Figure 10:**
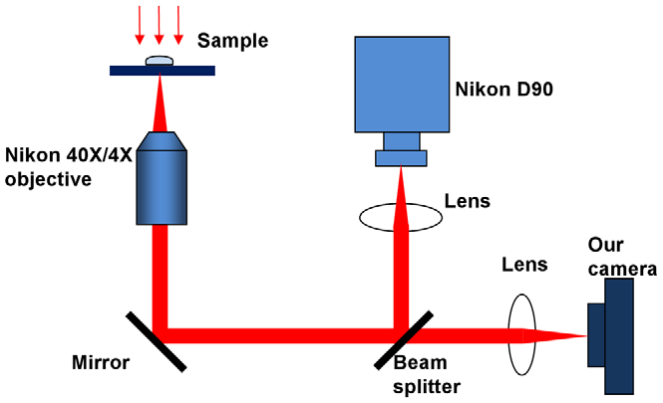
Experimental setup. A live *Daphina* (water flea) is placed in a small chamber on the stage of a microscope (Nikon ECLIPSE Ti). A commercial camera (Nikon D90) is used to obtain high pixel-count wide-field images while our camera is used for high-speed observation. Different objectives (4×, NA = 0.13 and 40×, NA = 0.74 respectively) are used to observe the beating heart and the flowing blood cells of the *Daphnia*.

The camera was installed on a commercial microscope (Nikon ECLIPSE Ti) as shown in Figure 10. The heart was observed with a 4×0.13 NA objective lens while the blood cells were observed with a 40×0.75 NA objective. Illumination was performed by a white light source from above the sample.

### Imaging modes

#### Basic operation

**Figure 11:**
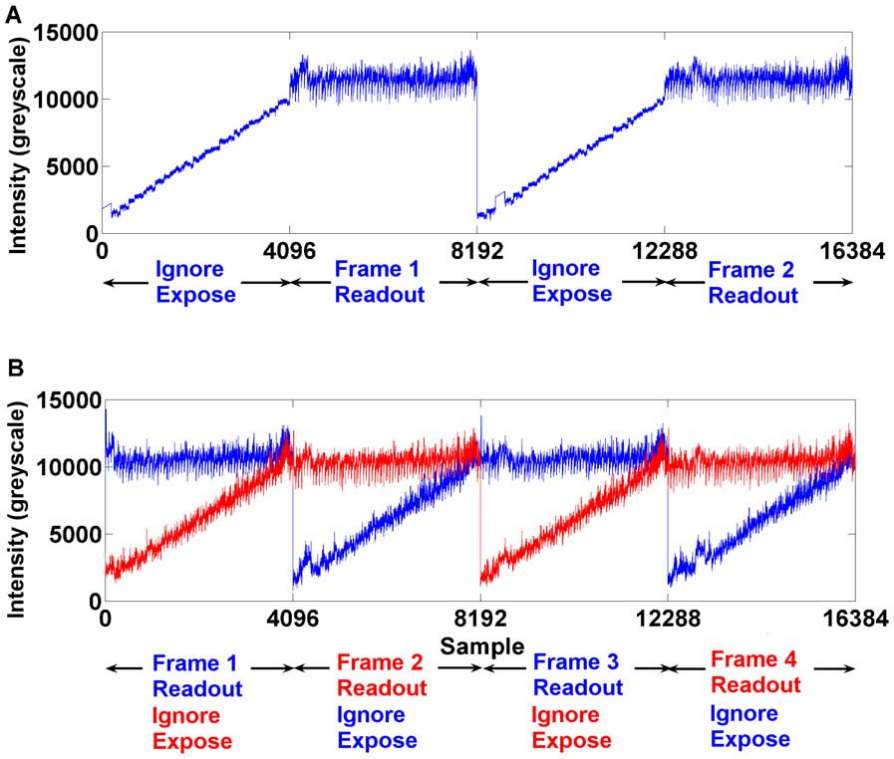
Example raw data traces. **A**) An example trace of the raw data obtained during readout of one channel. Although the sensor is first exposed and then read out sequentially, pixels are sampled continuously at a constant (synchronised) rate. During the exposure time samples are acquired but are simply discarded (marked ‘Ignore’), while during the readout time (marked ‘Read’) the sensor is blind and does not collect photons. **B**) Raw data obtained during readout in dual-channel mode. Exposure and readout alternate on two channels. This means the photodiode on each pixel is able to collecting photons continuously and simply alternate which storage capacitor it uses. In this way the frame rate is doubled and the dead-time can be virtually eliminated.

For continuous, synchronous, data acquisition we sample continuously and ignore the sampled data during exposure. One channel was used to do the exposure and readout sequentially. Pixels were sampled with 15 bits precision at a rate of 500 kS/s and the exposure time was set to 8.2 ms. The full, 4096 pixel, frame rate is therefore 61 fps. Figure 11A shows an example trace of the raw data acquired from one channel.

Note that the slopes seen during exposure simply show the discharging of the pixels during exposure due to the incident light. The dead-time is labelled ‘Readout’ (Figure 11A).

#### Dual channel sampling

In order to eliminate the dead-time, and avoid wasting photons during readout, we used two channels to do the exposure and readout in turn. The sampling rate was 500 kS/s. The exposure time was therefore set to be the same as the readout time (8.2 ms) to ensure there was no dead-time. Figure 11B shows the raw data acquired from the two channels at a frame rate of 122 fps.

#### *Daphnia* heartbeat

**Figure 12:**
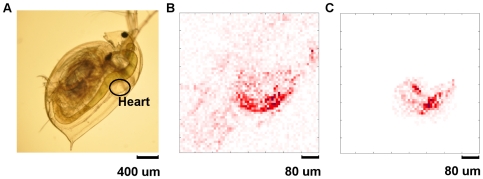
*Daphnia*'s heart. **A**) Image of a *Daphnia* in a chamber on the stage of a microscope (Nikon ECLIPSE Ti) taken with a commercial camera (Nikon D90). The beating heart is clearly observed (see supplementary material Movie S1). **B**) A differential image (obtained by subtracting adjacent frames) of the heart in full-frame mode (see supplementary material Movie S2). Image were acquired at 24.6 fps and captured simultaneously with a high-speed ROI. **C**) ROI acquired at high-speed (1572 fps). The size of the ROI is 512 pixels (see supplementary material Movie S3).

A live *Daphnia* was placed in a chamber and its beating heart (circled in Figure 12A) was imaged. To provide an overall view of the *Daphnia* a wide-field, high-pixel count movie was captured using a commercial SLR camera (Nikon D90) at 24 fps (Movie S1 see supplementary material).

#### High-speed ROI with low-speed full frame

The heart of the *Daphnia* was then imaged at high speed by selecting an ROI. In this mode the camera recorded an ROI (512 pixels) at 1572 fps, while simultaneously the whole frame (4096 pixels) was acquired at 24.6 fps. This was performed in single-channel mode at 1 MS/s with a short exposure (60 µs) as plenty of light was available.

A movie of the ROI was captured at 1,572 fps and played back at 25 fps, which recorded the beating of the heart at high-speed (Movie S3 see supplementary material). A second movie, showing the full frame, was captured at 24.5 fps and played back at 25 fps (Movie S2 – see supplementary material). Screenshots from both movies are shown in Figure 12B and Figure 12C.

The differential images in Movie S2 were generated by subtracting each frame from the previous frame. The changes in adjacent frames in Movie S3 are very subtle, however, due to the high imaging speed so the subtraction was performed over the previous 15 frames to produce the differential image.

Note that the fixed-pattern noise has not been removed from the raw images. This could be easily removed by subtracting a fixed, pre-recorded, background or adaptively by using double-sampling mode. However, it is not necessary to do so in this case because we are only interested in the differential images. Generating differential images subtracts out the fixed-pattern noise and clearly shows the moving edge of the heart.

**Figure 13:**
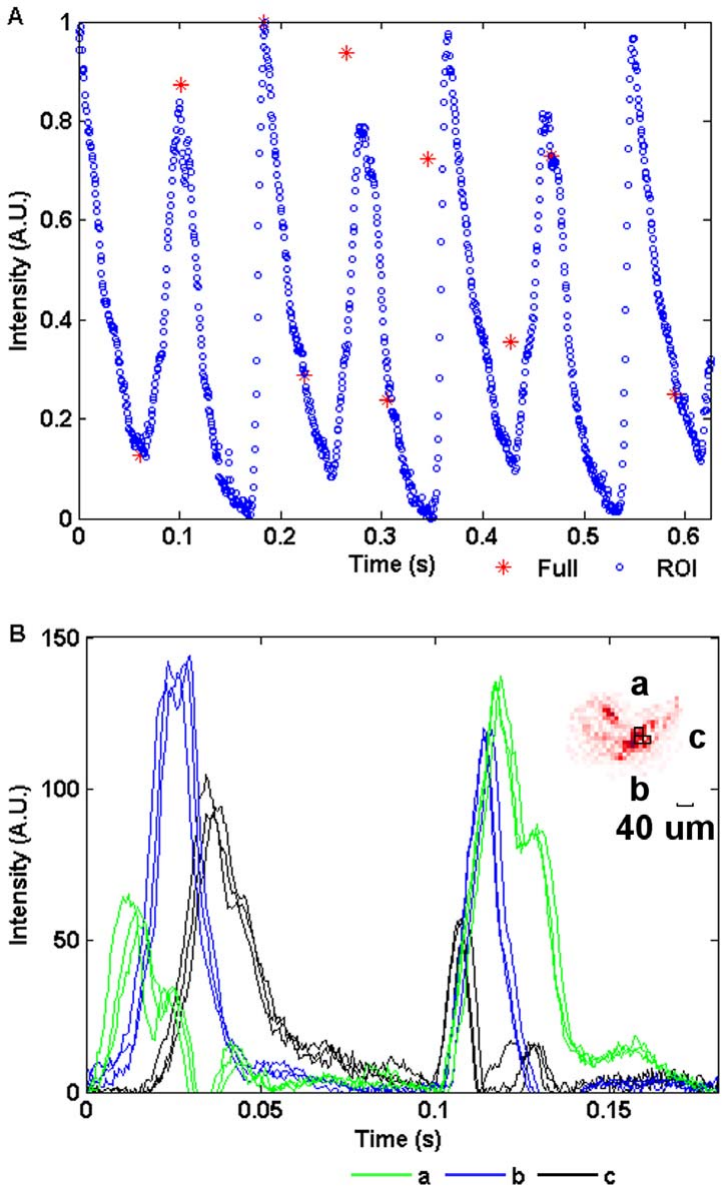
*Daphnia's* heartbeat. **A**) The mean value of each differential frame is plotted for both the full frame (*) and the ROI (o) image sequences. The sampling rate for the full-frame (24.56 fps) is too low to resolve the high-speed transients in the heartbeat but they can be seen clearly at 1572 fps. Note that the differential images show movement in the sample so each heartbeat results in a pair of spikes in this plot. The heart rate of the *Daphnia* is, therefore, approximate 5 Hz. **B**) The mean values of different regions (**a**, **b** and **c**, labeled in the top right figure of **B**) of the heart are compared and show the propogation of the wave across the heart muscle. Three continuous cycles of heartbeats have been overlayed in this figure to illustrate the consistency.

By calculating the mean greyscale of the heart the heart rate of the daphnia could be plotted (Figure 13A). Note that because the differential figures show movement in the sample there are two pulses for each heartbeat. While the full frame rate is too slow to show the heart beating (Figure 13A), the ROI trace clearly shows that the heart rate of this daphnia is approximately five beats per second and contains a fast upstroke followed by a slower downstroke. This allows for a detailed analysis of the heart dynamics that is essential in most quantitative cardiac imaging applications [20]. The movement of the heart muscle can also be illustrated by showing the mean greyscale in small regions within the ROI. The mean intensity of regions a, b and c (Figure 13B inset) are plotted in S13B and show the propagation of a contractile wave across the heart. Three sequential heartbeats have been overlayed in this figure to demonstrate their consistency.

**Figure 14:**
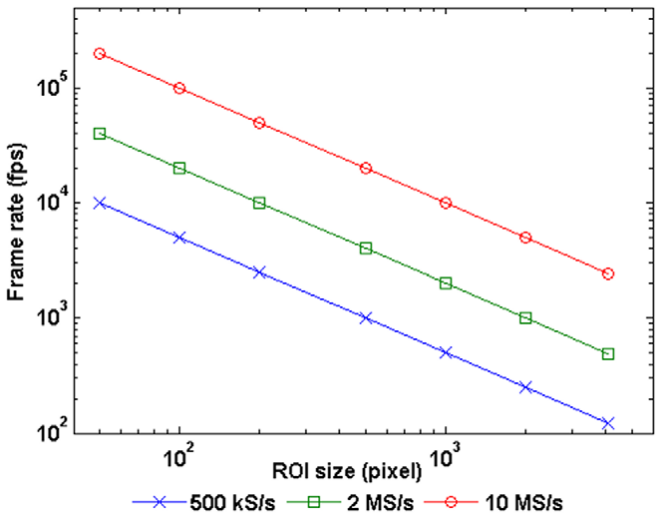
Possible frame-rates. Frame-rate versus ROI size in dual channel mode for different acquisition sample rates (MS/s per channel). The frame-rate can be increased by decreasing the size of the ROI or by using a faster acquisition (DAQ) card. We have used a 1 MS/s card (500 kS/s in Dual Channel Mode) although the CMOS sensor is capable of operating at 10 MS/s per channel.

The frame rate of the ROI is determined by its size (Figure 14). By selecting less pixels we can increase the frame rate arbitraily within the limits of the ADC card. The sample rate could also be further improved with a faster ADC card up to a limit of 20 MHz, or approximately 5,000 full frames per second. This limit is imposed by the bandwidth of the camera output amplifiers.

#### Tracking high-speed ROI with low-speed full-frame

**Figure 15:**
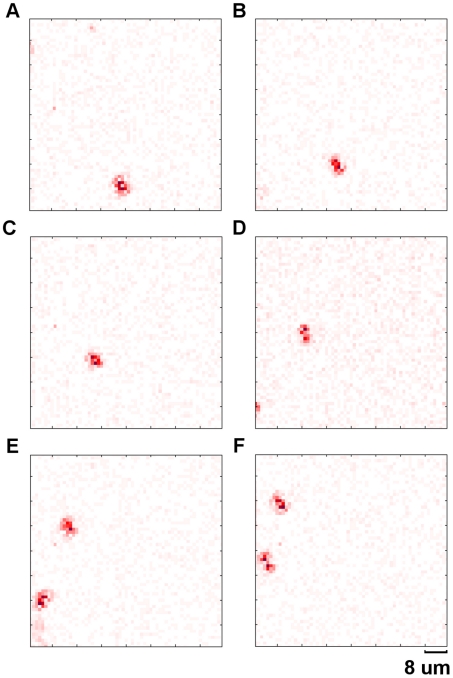
Blood cell flow. Sequence of differential frames showing individual cells flowing along blood vessels in a live *Daphnia*. These images were acquired in full frame mode at 24.6 fps. Frame 1 (**A**), 8 (**B**), 15(**C**), 22(**D**), 29(**E**) and 36(**F**). These differential images are obtained by subtracting adjacent frames. A second blood cell can be seen entering into the field of view in frame 22 (**D**) (see supplementary material Movie S4).

**Figure 16:**
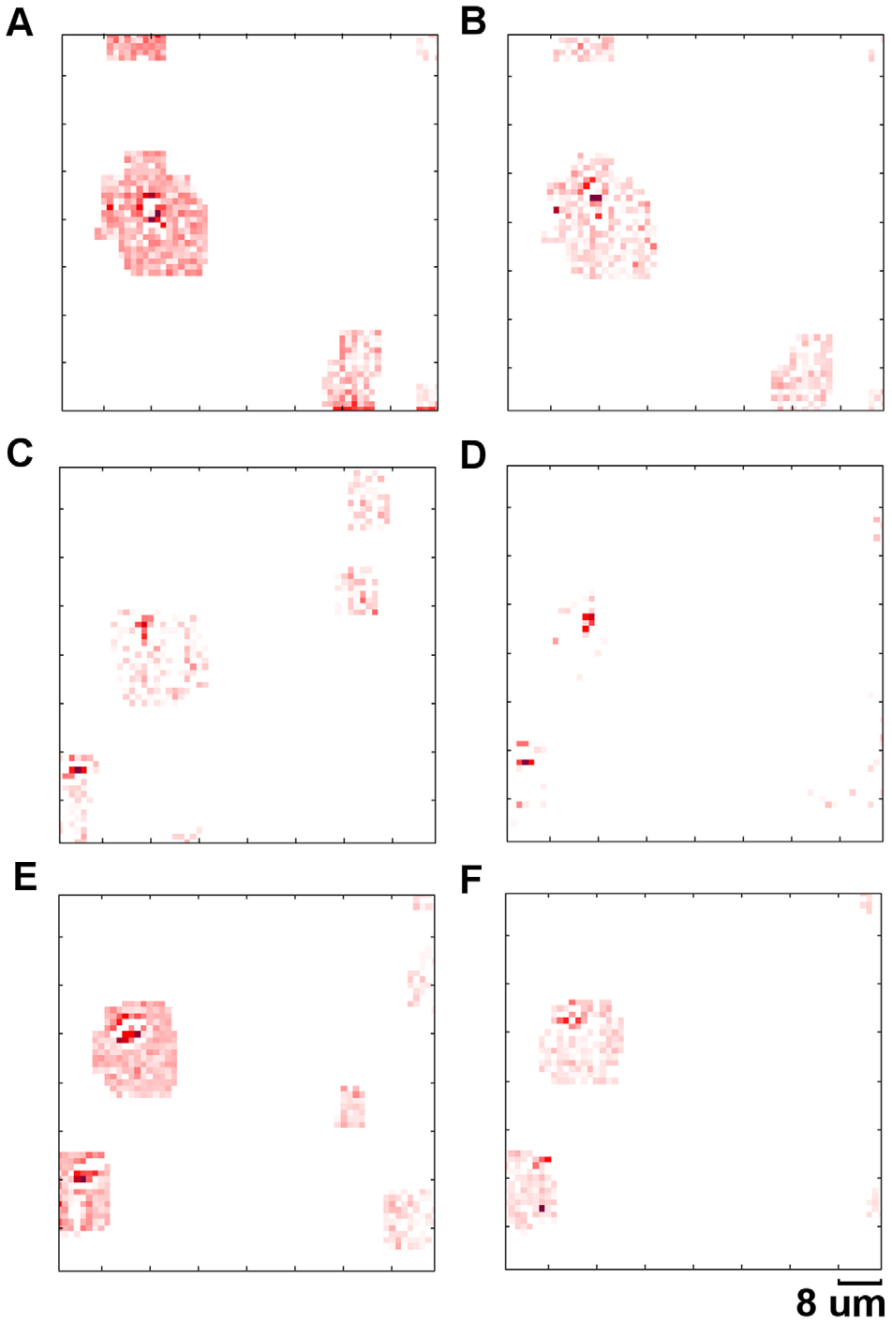
Blood cell flow - high-speed. Sequence of differential frames showing individual cells flowing along blood vessels in a live *Daphnia*. These images were acquired in ROI tracking mode at 1572 fps. Frame 1 (**A**), 101 (**B**), 201 (**C**), 301 (**D**), 401 (**E**) and 501 (**F**). The size of the ROI (red region) is 512 pixels. The locations of ROIs (red regions) are continuously updated in this mode to allow the moving blood cells to be tracked. The camera is able to automatically identify and image the second cell when it enters into the field of view (**E**), (see supplementary material Movie S5).

At higher magnification (40×0.75 NA) the blood cells can be seen flowing in the *Daphnia's* vascular system. The blood cells move fast and cannot be imaged with a fixed ROI. To monitor the movement of blood cells we enabled the tracking function of the camera and captured two sequences of images: one full frame sequence (Movie S4 – see supplementary material) and one tracking ROI sequence (Movie S5 – see supplementary material). Screenshots from both movies are shown in Figures 15 and 16.

In Movie S4 the red pixels show two blood cells flowing through the image rapidly. The full frame movie was captured at 24.6 fps and played back at 25 fps. The camera was used in tracking mode to update the ROIs automatically in Movie S5. This allowed the moving blood cells to be tracked continuously at high speed. Movie S5 was simultaneously captured at 1572.3 fps and played back at 25 fps. When the second cell enters the field of view the camera automatically detects it and is able to track both cells.

**Figure 17:**
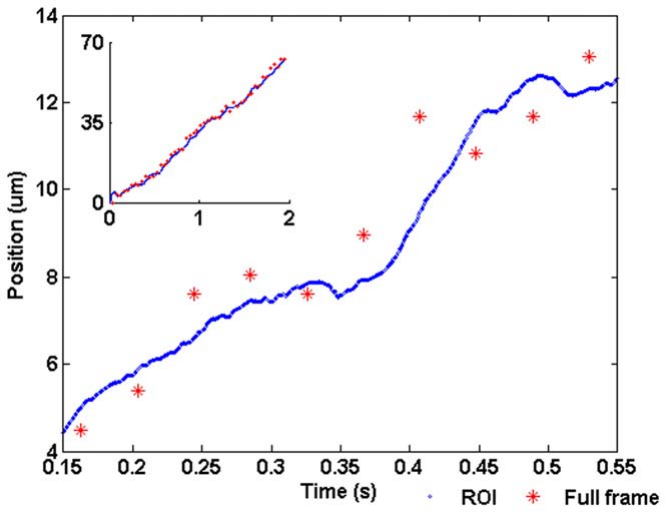
Blood cell position. The cell's position was sampled at 24.56 fps from the full-frame image sequence (*) and at 1572 fps from the ROI image sequence (o). The ROI curve has been smoothed to remove high frequency noise. The ROI data shows the pulsing of the blood cell that occurs with each heart beat. The inset demonstrates that the ROI is capable of tracking the blood cell for the full two seconds that it was within the camera's field-of-view without losing track of the target.

Figure 17 shows the parameterised position of the blood cell (r) along its trajectory against time. The cell's position was measured from both ROI and full frames. The ROI curve was smoothed to remove the high frequency noise. The pulsing of the cell is illustrated by the sharp slope in the plot. Although there is a latency before the ROI is updated, as discussed above, the ROI was able to cover the cell at all times. This demonstrates it is possible to observe a moving target at a high speed while simultaneously monitoring the background.

## Discussion

In this paper we have described a multifunction camera that can be operated in many different modes and is simple to reconfigure. Both the hardware and software of our system are modular and module replacement can be performed with minimal changes. The QSM-PC programming architecture and the feed-back control we have implemented allow the camera to be easily synchronized with other devices in an experimental rig.

While the overall bandwidth is low the camera can achieve respectable frame rates for an arbitrarily shaped region of interest that can be adaptively modified during acquisition. This reduces the volume of data considerably and relieves pressure on down-stream systems; making real-time data analysis easier to implement. This simple, low-bandwidth, system achieves high-speed ROIs (512 pixels at 1,572 fps) that are comparable with high specification commercial cameras. For example, MiCAM02-CMOS camera from SciMedia (92×80 pixels at 1,667 fps), DS-1x-16K5H camera from DALSA (128×128 pixels at 490 fps) and CardioCCD-SMQ camera from Redshirt Imaging (80×80 pixels at 2,000 fps). It has the added advantage of being able to dynamically reconfigure its ROIs in response to changes in the sample.

Although we have used a custom-made CMOS chip (A64P) other CMOS sensor chips could be used with minimal reconfiguration of the software if full access to the address bus is available. This could include CMOS based electrode arrays, which can also be used to monitor the electrical activity of both cardiac myocytes and networks of neurons.

The multiple channels per pixel available on our CMOS sensor mean that the dead-time during readout can be virtually eliminated by using two channels per pixel to alternate exposure and readout. This ensures all available photons are collected. Multiple channels also offer the possibility of removing fixed-pattern noise adaptively using double-sampling.

In addition to a basic operating mode we have implemented an ROI mode for high-speed imaging applications. This enables the camera to record images continuously at high speed and stream the data to disk for an indefinite time. The ROI can also be acquired simultaneously with full-frame images. This can allow, for example, low resolution images at high speed to be acquired at the same time as high resolution images at low speed. We have also implemented feed-back control to the camera, which allows the ROI to be determined from the full frame images. This means the ROIs can not only run at high speed but they can track moving targets.

One limitation of our system is that the latency between the command generation and updating is not consistent because it relies on the Windows operating system. To achieve a fixed latency the LabVIEW program could be transferred, with minor modifications, onto a Field-Programmable-Gate-Array (FPGA) data acquisition card.

In order to demonstrate the utility of our camera in a biological application the beating heart and flowing blood cells in a *Daphnia* were imaged. When monitoring the *Daphnia's* heart a reduced ROI was selected instead of the full frame. This increased the frame rate of the camera and allowed nuances of the heartbeat to be observed. The health of the heart and the subtle effects of toxic and therapeutic drugs on waves of contraction could be monitored at high speed in this way.

In a blood cell tracking experiment, we used the camera's ability to simultaneously acquire both in ROI and the full frame mode. The camera acquires a row during every time an ROI is read in order to slowly build up an image of the whole field. In this case we allocated the majority of the bandwidth (89%) to capture useful information (the ROI) and the remainder (11%) to check the full field. The ratio of this bandwidth allocation is arbitrary and depends on the demands of the application.

Although we didn't develop complex algorithms for this demonstration, any further developments can be easily integrated into the software. This could include more sophisticated post-processing of the images to improve the picture quality. Importantly no hardware changes are required to add functionality to the camera as this is achieved purely in software.

Finally, for applications that require very high-bandwidths, the camera system presented here could be transferred to larger sensors with faster hardware and faster data-links with relative ease.

In summary, we have developed a flexible, real-time, adaptive camera system based on a modular design that is capable of compressive sampling in a range of operating modes and demonstrated its use in a simple cardiac imaging experiment.

## Supporting Information

Movie S1 **Live** ***Daphnia*** **.**
(AVI)

Movie S2 **Full frame video of** ***Daphnia***'**s heartbeat.**
(AVI)

Movie S3 **Region-of-interest video of** ***Daphnia***'**s heartbeat.**
(AVI)

Movie S4 **Full frame video of** ***Daphnia***'**s blood cell movement.**
(AVI)

Movie S5 **Region-of-interest video of** ***Daphnia***'**s blood cell movement.**
(AVI)
